# *Klebsiella pneumoniae yggG* Gene Product: A Zinc-Dependent Metalloprotease

**DOI:** 10.3390/ijms12074441

**Published:** 2011-07-07

**Authors:** Chee Sian Kuan, Mun Teng Wong, Sy Bing Choi, Ching Ching Chang, Yoke Hiang Yee, Habibah A. Wahab, Yahaya Mohd Normi, Wei Cun See Too, Ling Ling Few

**Affiliations:** 1 School of Health Sciences, Health Campus, Universiti Sains Malaysia, 16150 Kubang Kerian, Kelantan, Malaysia; E-Mails: kcs10_psk046@student.usm.my (C.S.K.); wendywong84@gmail.com (M.T.W.); jinjinde_86@yahoo.com (C.C.C.); yee_hiang85@yahoo.com.ph (Y.H.Y.); stweicun@kb.usm.my (W.C.S.T.); 2 Pharmaceutical Design and Simulation (PhDS) Laboratory, School of Pharmaceutical Sciences, Universiti Sains Malaysia, 11800 Minden, Pulau Pinang, Malaysia; E-Mail: sybing@gmail.com; 3 Department of Cell and Molecular Biology, Faculty of Biotechnology and Biomolecular Sciences, Universiti Putra Malaysia, 43400 Serdang, Selangor, Malaysia; E-Mail: normi@biotech.upm.edu.my

**Keywords:** *Klebsiella pneumoniae* MGH 78578, yggG gene, KPN_03358, metalloprotease, HEXXH motif

## Abstract

*Klebsiella pneumoniae* causes neonatal sepsis and nosocomial infections. One of the strains, *K. pneumoniae* MGH 78578, shows high level of resistance to multiple microbial agents. In this study, domain family, amino acid sequence and topology analyses were performed on one of its hypothetical protein, YggG (KPN_03358). Structural bioinformatics approaches were used to predict the structure and functionality of YggG protein. The open reading frame (ORF) of *yggG*, which was a putative metalloprotease gene, was also cloned, expressed and characterized. The ORF was PCR amplified from *K. pneumoniae* MGH 78578 genomic DNA and cloned into a pET14-b vector for heterologous expression in *Escherichia coli*. The purified YggG protein was subsequently assayed for casein hydrolysis under different conditions. This protein was classified as peptidase M48 family and subclan gluzincin. It was predicted to contain one transmembrane domain by TMpred. Optimal protein expression was achieved by induction with 0.6 mM isopropyl thiogalactoside (IPTG) at 25 °C for six hours. YggG was purified as soluble protein and confirmed to be proteolytically active under the presence of 1.25 mM zinc acetate and showed optimum activity at 37 °C and pH 7.4. We confirmed for the first time that the *yggG* gene product is a zinc-dependent metalloprotease.

## 1. Introduction

*Klebsiella pneumoniae* is a Gram-negative, cylindrical rod shaped opportunistic pathogen that is found in the environment as well as a normal flora in mammalian mucosal surfaces like mouth, skin, and intestines. It is clinically the most important member of the family of Enterobacteriaceae that causes neonatal sepsis, nosocomial infections [1] and pneumonia [2]. Studies conducted in Asia estimated the incidence rate in elderly persons to be 15 to 40% [3,4] which is equal to or greater than that of *Haemophilus influenzae* [5].

*K. pneumoniae* strain MGH 78578 is one of the strains that shows high level of resistance to multiple antimicrobial agents including ampicillin, oxacillin, kanamycin, and chloramphenicol [6]. This strain was originally isolated from the sputum of a male patient in 1994 [6] and its genome has been sequenced by the Genome Sequencing Center of Washington University in Saint Louis in 2007. It was estimated that 20% of the total predicted open reading frames (ORFs) in the genome encode for hypothetical proteins, whose expressions and functions have not been proven experimentally. One of the hypothetical proteins is KPN_03358.

KPN_03358 has 231 residues of amino acids and codes for *yggG* gene. It was analyzed preliminarily using Uniprot. Uniprot [7] is an integrated database which performs retrieval of information from other databases such as metabolic database (KEGG) [8], protein–protein interaction (SPRING), family and motif databases (Pfam, InterProScan, PROSITE, HAMAP, *etc.*) homology database (SMR), dan Gene ontology (GO). Although *Klebsiella* YggG is classified as a putative uncharacterized protein, the result of sequence similarities annotation by Uniprot revealed that it belongs to peptidase M48 family. The gene ontologies (GO) indicated that the molecular function might be a hydrolase, metalloprotease or a protease based on the electronic annotation from InterPro scan database.

Metalloprotease, the most diverse of the six main types of proteases, has drawn much of our interest as it plays an important role in host-pathogen interactions by promoting enteropathogenicity, vascular permeability, host tissue damage and cytotoxicity [9]. Metalloproteases expressed by pathogens such as *Legionella pneumophila*, *Vibrio cholerae* and *Plasmodium vivax* involve in pathogenesis of the disease by degrading a wide range of host molecules [10–12]. The open reading frame of *yggG* gene (KPN_03358) from *K. pneumoniae* MGH 78578 was selected for cloning, expression and characterization in this study. This gene is highly conserved and its homologues could be found in various pathogenic microorganisms such as *Shigella dysenteriae*, *Shigella flexneri*, *Salmonella typhimurium* and *Salmonella enterica*. The gene was discovered in *Escherichia coli* when an open reading frame was found on the strand complementary to *speB* gene encoding agmatine ureohyrolase [13]. YggG is up regulated by heat shock and it interacts with Era protein, a membrane associated GTPase that is essential for *E. coli* viability [14]. Despite its proposed function as a heat shock protein [15] and its importance for cell response to stress [16], the protease activity of YggG has never been reported and thus, it is still being designated as a hypothetical metalloprotease. The *yggG* gene product from organisms other than *E. coli* has also never been investigated. Most of the proteases contain HEXXH site, however there are certain proteins with the HEXXH site that do not possess the protease activity [17]. Besides, previous expressions of proteases under the M48 family in *E. coli* are generally toxic to the host cells [18,19]. Thus, this study aims to heterologously express YggG and to confirm the proteolytic activity of purified YggG. In addition, computational bioinformatics approaches were also utilized in order to predict the possible structure and function of this YggG protein from *K. pneumonia* strain MGH 78578.

## 2. Results

### 2.1. Homology Modeling of YggG Protein and Model Assessment

Selected hypothetical protein YggG (KPN_03358) was subjected to BLAST (Basic Local Alignment Search Tool) search against NCBI non-redundant (NR) database. Putative conserved domain was detected as Peptidase M48 superfamily during the BLAST search. More than 100 hits were found with above the threshold of 0.001 Expected-value (E-value) and majority of them were either conserved hypothetical protein or metalloprotease. Subsequently, KPN_03358 underwent another round of BLAST search with PDB (Protein Data Bank) for potential template for homology modeling. Only one available PDB structure, 3C37 has the E-value above the threshold of 0.0001. 3C37 is the X-ray structure of putative Zn-dependent peptidase from *Geobacter sulfurreducens* with the length of 253 amino acid residues. It belongs to the M48 family of peptidase. Besides having similar length of amino acid residues, both KPN_03358 and 3C37 also share the same conserved domain. The sequence identity of KPN_03358 and 3C37 is 28% with the coverage of 88% of the whole sequence length. Hence, 3C37 was selected as the template for homology modeling of KPN_03358.

**Figure 1:**
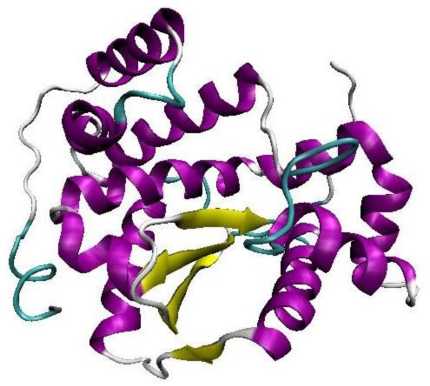
The model with the best Discrete Optimized Potential Energy (DOPE) scoring selected as our built model for KPN_03358. Alpha helix and beta sheet secondary structures are represented in purple and yellow, respectively. The graphic was generated using Visual Molecular Dynamics (VMD) visualization tool [21].

The best Discrete Optimized Potential Energy (DOPE) scoring built model was selected out of the 20 randomly generated models by MODELLER. In the 3 dimensional (3D) homology model of KPN_03358 (Figure 1), the four beta strands are located in the center of the structure and they are sandwiched in between two bundles of alpha helices (Figure 1). The conserved secondary structure regions found in the multiple sequence alignment are located in the center of the structure.

The best built model was then validated using PROCHECK [20] and the Ramachandran plot showed that 98.5% of the total residues fell within the most favorable and additional allowed regions. One residue however, fell within the disallowed region. Nevertheless, based on the Ramachandran plot, the model can be accepted as the best potential model representing KPN_03358 hypothetical protein.

### 2.2. Domain Family, Amino Acid and Membrane Topology Analyses of YggG Protein

**Figure 2:**
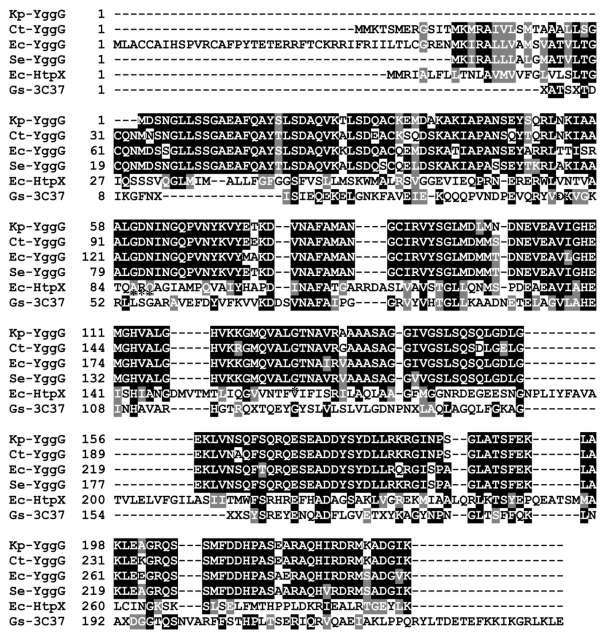
Amino acid sequence alignment of putative peptidase M48 family members of *Klebsiella pneumoniae* MGH 78578, Kp-YggG (YP_001336985); *Cronobacter turicensis*, Ct-YggG (YP_003211836); *Escherichia coli*, Ec-YggG (YP_854153); *Salmonella enterica*, Se-YggG (NP_461993); *E. coli* HtpX, Ec-HtpX (AAA62779); *Geobacter sulfurreducens* PCA, Gs-3C37 (3C37_A). GenBank accession numbers are given in parentheses. Asterisks indicate amino acids involved in zinc binding.

The ORF of *yggG* gene codes for a total of 231 amino acids with a calculated molecular weight of 24.7 kDa and a theoretical pI value of 5.76. Amino acids number 19 to 220 were aligned to the peptidase M48 family by Pfam sequence search. Figure 2 shows the amino acid sequence alignment between *K. pneumoniae* MGH 78578 YggG and its homologues from other species. There is one conserved putative domain found in all hits and is identified as HEXXH motif. The HEXXH motif and the third glutamic acid (55 amino acids C-terminal of the HEXXH motif) that are involved in the binding of zinc ion are marked with asterisks. *K. pneumoniae* YggG showed around 90% sequence identity with YggG proteins from *E. coli*, *S. enterica* and *Cronobacter turicensis*. In contrast, *K. pneumoniae* YggG showed only 18 and 19% sequence identities with *E. coli* HtpX [22,23] and *G. sulfurreducens* 3C37, respectively. Based on the identified conserved motif, the *K. pneumoniae* YggG protein can be classified under peptidase M48 family that belongs to subclan MA(E) or gluzincin of clan MA metalloprotease.

**Figure 3:**
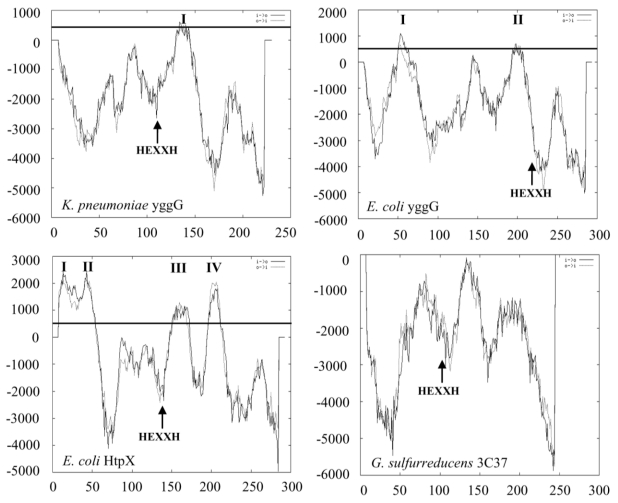
Comparison of transmembrane domains of *K. pneumoniae* YggG, *E. coli* YggG, *E. coli* HtpX and *G. sulfurreducens* 3C37 as predicted by TMpred. The horizontal line represents the level of hydrophobicity (score ≥ 500) that predicts membrane-spanning domains with high probability. Predicted transmembrane domains are indicated with Roman numerals. The position of metalloprotease motif (HEXXH) is also indicated. The X axis is the amino acid sequences of the enzyme and the Y axis is the hydrophobicity of the residues.

Figure 3 shows the membrane topology analysis of *K. pneumoniae* YggG by TMpred, which predicted a single membrane spanning domain from amino acid 125 to 146. The zinc binding motif (HEXXH and Glu-168) was predicted to be located in the cytosolic domain. In comparison, *E. coli* YggG, which is a membrane associated protein according to Huang *et al*. [15], contains two TMpred predicted transmembrane regions at amino acid number 3 to 20 (score = 1004) and number 152 to 170 (score = 702). In contrast, *E. coli* HtpX and *S. cerevisiae* Ste24p (Ste24p data not shown) were predicted to contain four and six transmembrane domains, respectively, their zinc binding motif was also predicted to be in the cytosolic domain. The hydrophobicity score of *K. pneumoniae* YggG predicted transmembrane domain was 601 (transmembrane helix score >500 predicts membranespanning domains with high probability) compared to transmembrane regions of HtpX and Ste24p with scores of up to 2000. As shown in Figure 3, no transmembrane domain was predicted for *G. sulfurreducens* 3C37 protein by TMpred. It is interesting to note that potential transmembrane hydrophobic region was not predicted in *K. pneumoniae* YggG by other programs like SOSUI [24], hmmtop [25,26], TMHMM [27] and PhDhtm [28].

### 2.3. Cloning and Heterologous Expression of YggG in E. coli

**Figure 4:**
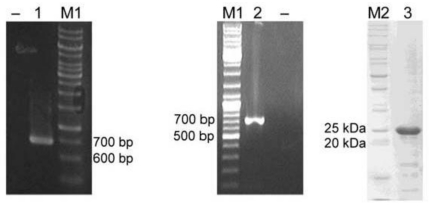
PCR amplification and expression of *K. pneumoniae* MGH 78578 *yggG* ORF. A 696 bp PCR product was amplified from genomic DNA (lane 1) or cDNA (lane 2). The PCR product from lane 1 was purified and cloned into a pET-14b vector for protein expression “–”lanes indicate no-template controls. Lane 3: Purified *K. pneumoniae* YggG protein on 12% SDS-PAGE. M1: GeneRuler 1 kbp DNA ladder (Fermentas); M2: BenchMark^™^ protein ladder.

A 696 bp ORF of *yggG* gene was amplified from *K. pneumoniae* MGH 78578 genomic DNA (Figure 4, lane 1) by PCR and it was cloned into a pET14-b vector for the expression of YggG as a 6× histidine tagged (His_6_-tagged) protein in *E. coli*. The optimal induction time and temperature were 6 h and 25 °C. More than 80% of the His_6_-tagged YggG was present as soluble fraction under the purification procedure used in this study. The histidine tag was removed in the final step and the typical yield of purified YggG (Figure 4, lane 3) per liter culture was around five milligrams.

### 2.4. Detection of yggG mRNA Expression in K. pneumoniae

The *yggG* mRNA was detected by RT-PCR as shown in Figure 4 (lane 2). A PCR product corresponding to the size of *yggG* ORF (696 bp) was successfully amplified from *K. pneumoniae* cDNAs. The result confirms that *yggG* is not a pseudogene.

### 2.5. Proteolytic Activity of Purified K. pneumoniae YggG

**Figure 5:**
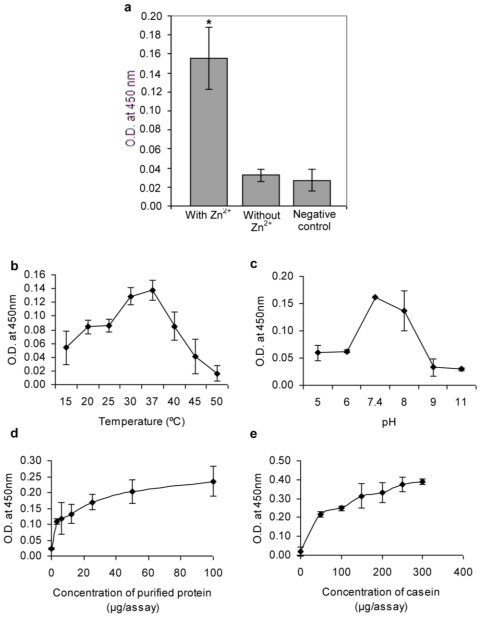
Effects of Zn^2+^ (**a**), temperature (**b**), pH (**c**), enzyme concentration (**d**), and substrate concentration (**e**) on the proteolytic activity of *K. pneumoniae* YggG. The protease activity was determined as the rate of succinylated casein hydrolysis. All the reactions were performed in triplicate and the data were plotted as their mean ± standard deviation. Asterisk indicates statistically significant difference (*p* < 0.05).

Proteolytic activity of purified YggG was confirmed by casein hydrolysis assay. As shown in Figure 5a, five folds higher activity was detected in the reaction containing 1.25 mM zinc acetate compared to reactions without zinc and negative control. There was no significant activity without the presence of zinc ion (*p* < 0.05). YggG showed optimum proteolysis at 37 °C and pH 7.4 (Figure 5b,c). The proteolytic activity of YggG increased hyperbolically with increasing enzyme and substrate concentrations and reached its maximum at about 100 μg/assay of YggG protein and 300 μg/assay of casein (Figure 5d,e).

## 3. Discussion

In this study, the *yggG* ORF encoding a putative metalloprotease in *K. pneumoniae* was cloned and expressed in *E. coli* as 6× histidine fusion protein. The protein was purified to apparent homogeneity and confirmed to be catalytically active under the presence of zinc ion. Protein family database search predicted YggG as a metalloprotease from the peptidase M48 family. The conserved HEXXH motif and a third glutamic acid residue responsible for metal binding were identified. Over the past decade, several microbial metalloproteases in the peptidase M48 family have been cloned and characterized, most prominent examples are Ste24p from *S. cerevisiae* [29], HtpX from *E. coli* [22] and HtpX-like heat shock metalloprotease from an unknown organism related to *Methylobacillus flagellatus* [19]. The HEXXH motif in this group of protease is critical for their catalytic activity since the proteolytic activities of Ste24p and HtpX were lost when the motif was mutated [29,30].

*K. pneumoniae* YggG has a smaller molecular mass (~25 kDa) compared to Ste24p (52.3 kDa) and HtpX (32 kDa) proteins. Transmembrane helices prediction with TMpred (Figure 3) also showed contrasting topologies between these proteins. The distinctive features of *K. pneumoniae* YggG suggest that this protein could be functionally different from Ste24p or HtpX. Comparison with its putative ortholog from *E. coli* [13,15] also revealed some differences in terms of the number of putative transmembrane domain and solubility. *E. coli* YggG was predicted to have an extra transmembrane domain at the N-terminus (Figure 3) and it was reported to be membrane bound [15] whereas we have successfully expressed the *K. pneumoniae* YggG as a soluble protein.

Heterologous expressions of metalloproteases usually encounter the problem of low solubility due to their incorporation into the membrane of the host organism and their associations with insoluble inclusion bodies. Laborious refolding step was required to obtain the proteases in their active and stable forms [19,23,31]. The full length and 21 amino acids N-terminal truncated versions of YggG protein from *E. coli* were also membrane-associated [15]. On the contrary, the *K. pneumoniae* YggG produced in this study was soluble when expressed in *E. coli*. Recently, an astacin metalloprotease from a parasitic nematode, *Steinernema carpocapsae* was also successfully expressed as a soluble protein in *E. coli* [32]. Expression of *K. pneumoniae* YggG as a soluble protein was in agreement with the results from topology analysis that predicted a single weak hydrophobic transmembrane domain in this protein.

Most metalloproteases are characterized by a catalytic zinc ion although in some enzymes, manganese, cobalt, nickel or even copper ions can also undertake this function [33]. The purified *K. pneumoniae* YggG exhibited significant casein proteolytic activity when zinc ion was supplied as a metal cofactor. The presence of a zinc-binding motif, HEXXH, in this enzyme suggests that the catalytic activity of YggG is modulated by zinc through its binding to this site [22,23,30]. Specific point mutation on this motif can be done to confirm the importance of zinc binding to YggG’s activity.

The sequence of *yggG* ORF cloned in this study was based on the completed GenBank genome sequence of *K. pneumoniae* strain MGH 78578. Recently, completed genome sequences of N_2_-fixing *K. pneumoniae* 342 (KPK_0741) [34] and liver abscess and meningitis causing *K. pneumoniae* NTUH-K2044 (KP1_4646) [35] strains have been published. The deduced YggG protein sequences from these two strains have an additional stretch of 21 amino acids that are highly similar to the *E. coli* YggG protein. This stretch of amino acids would also constitute a putative hydrophobic transmembrane domain. However, based on bioinformatics analysis and molecular mass of immunodetected *E. coli* YggG protein, it was suggested that the first 17 to 19 amino acids were removed in the mature YggG protein [15]. Therefore, the catalytically active YggG of *K. pneumonia* MGH 78578 produced in this study has a high likelihood of resembling the mature YggG in the cell.

## 4. Experimental Section

### 4.1. Computational Methodology for Homology Modelling

YggG protein sequence was subjected to a series of BLAST search against non-redundant database (NR) and PDB for template selection. Its structure prediction was done using MODELLER 9v8 [36]. 20 models were generated randomly and the best Discrete Optimized Potential Energy (DOPE) scoring model was selected. Verification of the built model was done using PROCHECK.

The domain family analysis was performed using Pfam protein families database [37]. ClustalW2 [38] was used to perform the multiple sequence alignment with related metalloproteases. Transmembrane region of YggG protein was predicted and compared to other metalloproteases under the same peptidase M48 family by using TMpred [39].

### 4.2. Bacterial Strain, Growth and Culture Conditions

*Klebsiella pneumoniae* subsp. *pneumoniae* MGH 78578 (ATCC number 700721) was used for this study. The bacterial strain was routinely cultured in Luria-Bertani [18] medium at 37 °C.

### 4.3. Total Genomic DNA and RNA Extractions from *K. pneumoniae* MGH 78578

Genomic DNA and total RNA were isolated from a five mililiter overnight culture of *K. pneumoniae* MGH 78578 using QIAmp DNA Mini Kit (Qiagen) and RNeasy Mini Kit (Qiagen), respectively, according to the manufacturer’s protocol. The integrity and size distribution of total purified RNA was visualized by ethidium bromide staining after electrophoresis on a 1% agarose gel.

### 4.4. PCR Cloning of *K. pneumoniae yggG* Open Reading Frame

The *yggG* ORF was PCR amplified from *K. pneumoniae* genomic DNA in a 50 μL reaction consisted of 10× Thermopol buffer (New England Biolabs), 1 μM each of forward (5′-GAATTCCATATGGACTCCAACGGTCTGCTCAGC-3′) and reverse (5′-CGCGGATCCTTATTTAATGCCGTCGGCCTTCATGC-3′) primers, 5 mM dNTPs, 1 unit of Taq polymerase and 100 ng genomic DNA. The PCR was performed for 30 cycles of 95 °C for 30 s, 66 °C for 30 s and 72 °C for 60 s.

The PCR product was gel purified by Qiaquick gel extraction kit (Qiagen), digested with *Nde*I and *BamH*I (New England Biolabs) and ligated into a pET-14b vector (Novagen) precut with the same restriction enzymes. The resulting pET-14b-yggG was confirmed by sequencing.

### 4.5. Expression and Purification of YggG Protein

For protein expression, the pET-14b-yggG plasmid was transformed into the *E. coli* BL21 (DE3) strain. The culture was grown in LB medium (with 100 μg/mL ampicillin) at 37 °C, 200 rpm to an OD_600 nm_ of 1.8. Subsequently, the expression of 6 × histidine tag YggG was induced with 0.6 mM isopropyl thiogalactoside (IPTG) After the induction period, the cells were pelleted and re-suspended in 5 mL pre-cooled buffer (50 mM Tris-HCI, pH 7.2, 300 mM sodium chloride and 10 mM imidazole). The cells were then sonicated and centrifuged at 2000 × g for 20 min. Ni^2+^-NTA resin (Qiagen) was added to the supernatant and binding of the His-YggG was carried out by gentle mixing for 2 h. After the binding step, the resin was spun down at 440 × g for 5 min and the supernatant was discarded. The resin was washed eight times with 10 mL of the same buffer for 30 min. After washing, the resin was resuspended in 3 mL of buffer and six NIH units of bovine thrombin (MP Biomedicals) were added to cleave off the His-tag from the fusion protein. The purified protein was eluted as un-tagged YggG after overnight thrombin cleavage and quantified by using Bradford reagent (Bio-Rad).

### 4.6. Reverse Transcription PCR of *yggG* Gene

RevertAid H Minus first strand cDNA synthesis kit (Fermentas) was used to synthesize the cDNA from the extracted total RNA. One microgram of total RNA was mixed with 0.2 μg random hexamer primer and DEPC-treated water. Subsequently, the mixture was preheated at 70 °C for 5 min, chilled on ice and followed by the addition of 4 μL of 5 × RT buffer, 1 mM of dNTP mix and 20 units of Ribolock Ribonuclease inhibitor. The mixture was incubated at 25 °C for 5 min followed by incubation at 37 °C for another 5 min. 200 units of Revertaid H Minus M-MuLVRT was added to make up a total volume of 20 μL. The mixture was then incubated at 42 °C for 1 h and heated at 70 °C for 10 min for the termination of the reverse transcription process. PCR was performed as described above except that the template was replaced with one microliter of cDNA.

### 4.7. Casein Hydrolysis Assay

The protease activity of the purified YggG protein was measured by casein hydrolysis assay using a QuantiCleave Protease Assay Kit (Pierce). Briefly, 100 μL of succinylated casein substrate solution (2 mg/mL in assay buffer: 40 mM disodium hydrogen orthophosphate, pH 7.4) were added into one set of microtiter plate wells. Another duplicate set of wells were added with the assay buffer to serve as blanks. Forty microliters of purified YggG (1 mg/mL) were added into both the substrate and blank wells and incubated at 37 °C for 150 min. Subsequently, 100 μL of diluted trinitrobenzene sulfonic acid (TNBSA), 0.033% (w/v) were added to each well and incubated for 35 min at 37 °C. The absorbance at 450 nm was determined for every well and the proteolytic activity was represented by the change in absorbance (ΔA_450_), which was calculated by subtracting the absorbance of the blank from that of the corresponding casein well. Data from a triplicate experiment were analyzed with one way ANOVA using SPSS version 15.0. The level of significance was set at *p* = 0.05. The assay was also repeated with assay buffer containing 1.25 mM zinc acetate. Reaction without purified YggG was used as the negative control.

The effects of purified YggG (0 to 100 μg/assay) and substrate (0 to 300 μg/assay) concentrations on the protease activity were also determined under the presence of 1.25 mM zinc acetate and the assay conditions as described above.

### 4.8. Determination of Temperature and pH Optima of the Activity of Purified YggG

Forty micrograms of purified protein and 100 μg of succinylated casein were used per assay for the determination of optimum temperature and pH for YggG casein hydrolysis activity. The buffers used were 100 mM acetate buffer for pH 5.0 and 6.0, and 40 mM disodium hydrogen orthophosphate buffer for pH 7.4, 8.0, 9.0 and 11.0. The effect of temperature on the protease activity of YggG was studied by incubating the standard reaction mixture at temperatures ranging from 15 °C to 50 °C for 150 min.

## 5. Conclusions

Here, we have presented the predicted model as well as described the cloning, expression, purification and characterization of YggG metalloprotease from *K. pneumoniae* MGH 78578. This enzyme was soluble in *E. coli* and required zinc as cofactor for catalysis. The soluble and active YggG protein can promote further biochemical studies such as the identification of natural substrate(s) and the search for inhibitors of this enzyme [40]. It is interesting to see whether *yggG* knockout of *K. pneumoniae* strain is also not lethal as in the case of *E. coli* [14] and whether it would have any effect on the virulence of *K. pneumoniae* similar to the previously reported peptidase M48 family of *Porphyromonas gingivalis* [41]. In addition, the feasibility of YggG heterologous expression in *E. coli* is hoped to encourage structural studies of this enzyme.
